# MicroRNA‐34c suppresses proliferation of vascular smooth muscle cell via modulating high mobility group box protein 1

**DOI:** 10.1002/jcla.23293

**Published:** 2020-03-10

**Authors:** Li‐Bo Chen, Zhe An, Hai‐Kuo Zheng, Xin‐Peng Wang, Rui‐Ting Shan, Cui‐Ying Mao, Wen‐Qi Zhang

**Affiliations:** ^1^ Department of Ultrasound China‐Japan Union Hospital of Jilin University Changchun China; ^2^ Department of Cardiovascular China‐Japan Union Hospital of Jilin University Changchun China

**Keywords:** atherosclerosis, high glucose, high mobility group box protein1, MicroRNA‐34c, proliferation, vascular smooth muscle cell

## Abstract

**Background:**

Atherosclerosis is the most frequent pathological process that causes cardiovascular diseases.

**Objective:**

The present study aimed to confirm miRNAs associated with atherosclerosis and explore the molecular mechanism of miR‐34c and its target high mobility group box protein 1 (HMGB1) in the control of growth of smooth muscle cells in the development of atherosclerosis.

**Methods:**

Real‐time PCR was firstly performed to confirm miRNA correlation with atherosclerosis, and computational analysis and luciferase assay were performed to explore the target of miR‐34c, Western blot, and real‐time PCR were also utilized to reveal the effect of whether high glucose (HG) and miR‐34c affect miR‐34c, HMGB1 levels, NF‐κB p65 and TNF‐α levels, and the role of miR‐34c on vascular smooth muscle cells (VSMCs) viability induced by HG. Students' unpaired *t* test was performed to compare data between two groups.

**Results:**

MiR‐34c level was associated with atherosclerosis with different expression between VSMCs treated with high glucose or normal VSMCs. Then, HMGB1 is a virtual target of miR‐34c with predicted binding site resided in HMGB1 3′UTR and further verified by that miR‐34c remarkably reduced luciferase activity of wild HMGB1 3′UTR under a concentration‐dependent fashion, and miR‐34c cannot influence luciferase activity of mutant HMGB1 3′UTR.

**Conclusions:**

The results suggested miR‐34c might be a novel therapeutic strategy in the management of atherosclerosis by suppressing the expression of HGMB1 and its downstream effectors.

## 1 INTRODUCTION

Atherosclerosis is the most frequent pathological process that causes cardiovascular diseases (CVD), a disease of medium‐ and large‐sized arteries that is featured by a development of atherosclerotic plaques made up of build‐up modified lipids, calcified regions, necrotic cores, inflamed foam cells, leukocytes, endothelial cells (ECs), and smooth muscle cells (SMCs).^1^ These characteristics of atherosclerotic plaques reveal that atherosclerosis is a complex disorder, and this process is associated with a variety of factors of the immune, metabolic, and vascular systems. The proliferation of vascular smooth muscle cells (VSMCs) is a key step of atherosclerosis.^2^ One mediator in formation of lesion in humans may be focal build‐up of intimal VSMCs.^3^ However, there is still debate in the accurate role of VSMCs in atherosclerosis.^4^ In early stage of atherosclerosis, VSMCs may be a contributor to the formation of the atheroma through the synthesis of matrix molecules necessary for the retention of lipoproteins and through the generation of pro‐inflammatory mediators including vascular cell adhesion molecule and monocyte chemoattractant protein 1.^3^

As a chronic inflammatory disorder, atherosclerosis is associated with complicated interactions among endothelial cells, T lymphocytes, macrophages, and multiple cytokines. High mobility group box protein 1 (HMGB1) is a pro‐inflammatory mediator that upregulates cytokines, chemokines, and adhesion molecules, which, in turn, enhance infiltration of macrophage and cause atherosclerosis.^5^ Moreover, it has been shown that blockade of HMGB1 results in inhibition of vascular inflammation and alleviation of atherosclerosis in ApoE^−/−^ mice.^6^ The bioinformatics of Target‐Scan identified that HMGB1 is a potential target for miR‐24 among these target genes. It is also identified as an extracellular trigger to mediate immune response to injury and inflammation.^7^ The key function ofHMGB1 in the activation of pro‐inflammatory signaling to a variety of cells was widely studied.^7^ Regarding vascular disorders, it is shown that atherosclerotic lesions have increased levels of HMGB1.^8^ Moreover, earlier study of our laboratory revealed that VSMC proliferation and migration in vitro could be significantly mediated by HMGB1.^9^

Representing small non‐coding sequences of RNA, miRNAs are expressed in the genome of various organisms including metazoans, viruses, and plants. Since their original discovery as a developmental modulator in the nematode *Caenorhabditis elegans*, miRNAs have now been demonstrated to have critical roles in modulation of gene expression in majority of organisms.^10^ MiRNAs bind to target mRNAs to play regulatory role at post‐transcriptional level, rendering a common mechanism for modulation of gene expression. Over 1.500 miRNAs have been identified in the human genome so far.^11^ It is estimated by computational studies that miRNAs can regulate over 60% of total genes.^12^ Consequently, these small endogenous silencers have appeared as epigenetic modulators of various biological processes including cholesterol metabolism, angiogenesis, cell proliferation, and development.^13^, ^14^, ^15^ To date, several miRNAs such as miR‐370, miR‐26, miR‐758, miR‐106, miR‐33, and miR‐122 have been identified to modulate metabolism of lipid as well as atherosclerosis progression and regression.^16^

High mobility group box protein 1 has been demonstrated to be associated with the pathogenesis of atherosclerosis, and it has been shown that dysregulation of miR‐34c is involved in the development of atherosclerosis.^17^, ^18^, ^19^ In the online miRNA database, HMGB1 was also identified as a possible target of miR‐34c. The present study aimed to explore the correlation between the HMGB1 and miR‐34c and studied their roles in the controls of high glucose‐induced apoptosis of smooth muscle cells.

## 2 MATERIALS AND METHODS

### 2.1 Cell culture and transfection

RPMI 1640 (Gibco) with 15% FBS (fetal bovine serum; Gibco), 100 μg/mL streptomycin (Sigma), and 100 U mL/L penicillin (Sigma) was utilized to incubate VSMCs under an atmosphere with 5% CO2/95% air at 37°C. Twelve hours later, the VSMCs were seeded into growth medium (Invitrogen) without antibiotic in 48‐well plate. When the cell grown to 80% confluence, transfection was performed after cell seeding for 24 hours using Lipofectamine 2000 (Invitrogen) following the protocol indicated by manufacturer. Three independent experiments were carried out.

### 2.2 VSMC proliferation assay

Firstly, the cells were cultured into 48‐well plates at a final density of 5 × 10^3^/100 mL/well, and maintained for 12 hours. Bromodeoxyuridine (BrdU) label at a dilution of 1:10 000 was utilized to treat the medium, and the cells were incubated for additional 16 hours. BrdU cell proliferation assay kit (Calbiochem) was utilized to detect the proliferation of cells, and GloMax‐96 Microplate luminometer (Promega) was also utilized to detect the proliferation based on the wavelength of 450 nm in accordance with the manufacturer's manual. All tests were performed three times.

### 2.3 Enzyme‐linked immunosorbent assay

Vascular smooth muscle cells supernatants were collected from various groups 48 hours post‐treatment with glucose, and enzyme‐linked immunosorbent assay (ELISA) kit (R&D Systems) was utilized to detect the cytokines (NF‐kB and TNF‐α) concentrations based on suppier's recommendation. All results were shown as pictograms/milliliter.

### 2.4 RNA isolation and real‐time PCR

Trizol^®^ Reagent (Invitrogen) was utilized to isolate total RNA from tissue samples and VSMCs based on protocol indicated by the manufacturer. UV‐Vis spectrophotometer UV‐1800 (Shimadzu) was used to examine the concentration of RNA. The PrimerScript RT reagent kit (Takara) was utilized to carry out DNA synthesis from RNA extracted followed guideline by the supplier. ABI 7500 Real‐Time PCR System (Applied Biosystems) with SYBR Premix Ex TaqTM II (Takara) was performed qPCR (quantitative real‐time PCR) following manufacturer's recommendation. U6 and GAPDH were served as the internal control. ABI 7500 Software 2.04 from Applied Biosystems with $2^{-\Delta \Delta C_{t}}$ method was utilized to calculate the relative expression of miR‐34c and HMGB1 mRNA.^20^ Three independent experiments were carried out.

### 2.5 Luciferase assay

PCR was performed to amplify fragment of HMGB1 3′UTR, and the PCR products were inserted into pMIR‐Report (Promega), and DNA sequencing was performed to confirm cDNA sequence and synthesized HMGB1 3′UTR forward and reverse DNA oligos contained miR‐34c potential binding sites, and inserted into the vector (Promega), then in order to perform mutation analysis, synthesized mutations located on miR‐34c binding site located within HMGB1 3′UTR and inserted as above. VSMCs were seeded into 48‐well plates and transfected with 50 nmol/L miR‐34c mimic, 50 ng psiCHECK2 vectors using Lipofectamine 2000 (Life Technologies). Then, dual‐luciferase reporter assay system (Promega) was utilized to determine luciferase activity of firefly and renilla 48 hours post‐transfection. All tests were carried out three times.

### 2.6 Western blot analysis

For Western blot analysis, RIPA buffer (Upstate Biotechnology) was utilized to lyze the cells 48 hours after transfection. The lysates were centrifuged at 5000 *g* for 15 minutes at 4°C, and the protein assay reagents (Bio‐Rad Laboratories) were utilized to detect the content of the protein following the instruction of the supplier. 12% SDS‐PAGE was utilized to separate the 20 mg total protein (20 mg) and then electro‐transferred to a pure nitrocellulose membrane (Bio‐Rad Laboratories), following by treating with Odyssey^®^ Blocking Buffer (LI‐COR Biosciences) containing 3% BSA at room temperature for 60 minutes. And the primary antibody against HMGB1 (1:3000, Cell Signaling Technology) was utilized to treat the membranes overnight at 4°C, and the internal control was chosen the β‐actin (1:10 000, Cell Signaling Technology, Inc) to normalize the values. And then diluted HRP‐conjugated IRDye^®^ 680LT Goat Anti‐Mouse lgG or IRDye 680LT Goat Anti‐Rabbit lgG (LI‐COR Inc) was utilized to incubate membrane at room temperature for 60 minutes. Odyssey CLx imaging system, model: Ody‐3086 (LI‐COR Inc), was used to scan the membrane were utilized to identify the immuno‐reactive bands in accordance with the manufacturer's instruction.

### 2.7 Statistical analysis

Prism 5.0 (GraphPad Software Inc) was utilized to conduct statistical analysis of data, and Students' unpaired *t* test was performed to compare data between two groups. *P* value <.05 was considered as statistical significant.

## 3 RESULTS

### 3.1 VSMC viability was interfered by glucose

**Figure 1:**
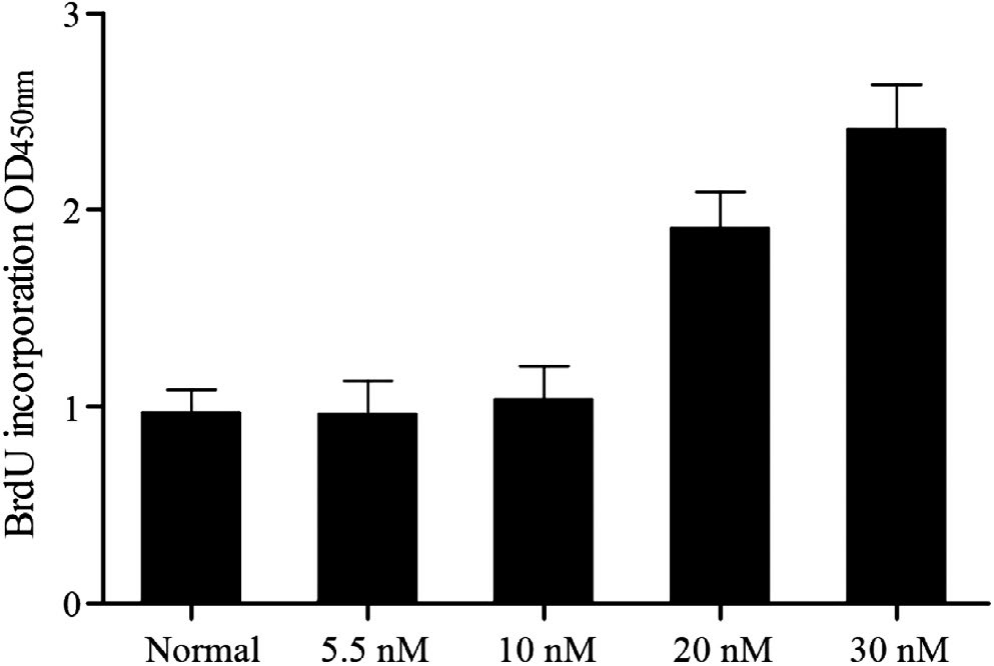
VSMCs treated with different dose of glucose (5.5, 10, 20, 30 nmol/L), only high glucose (≥20 nmol/L) enhanced proliferation of VSMCs

Vascular smooth muscle cells were isolated, cultured in RPMI 1640 medium, and treated with different dose of glucose (5.5, 10, 20, 30 nmol/L). BrdU cell proliferation assay kit was utilized to detect the effect of glucose on the proliferation of cells. As shown in Figure 1, treating with glucose had no obvious effect on VSMC proliferation at concentrations of 5.5 and 10 mmol/L compared with normal control, when the concentrations of glucose up to 20 nmol/L, an evidently higher cell proliferation ability related to normal control was observed; furthermore, the promotion effect of 30 nmol/L glucose was much stronger than 20 nmol/L glucose, suggested that high dose of glucose (≥20 nmol/L) triggered VSMCs proliferation.

### 3.2 MiRNAs differentially expressed in different treatments cells

**Figure 2:**
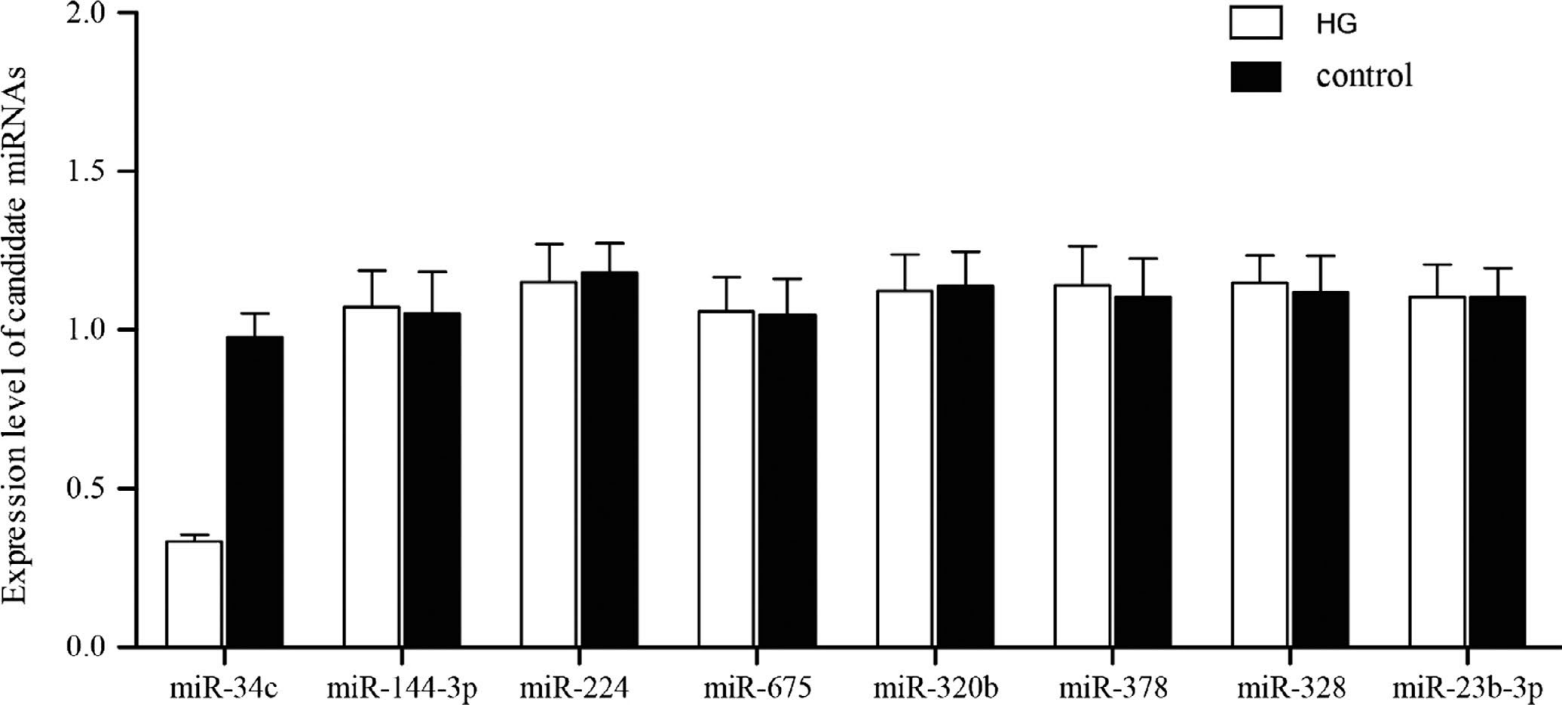
Real‐time PCR was performed to detect eight miRNAs (miR‐34c, miR‐144‐3p, miR‐224‐SNP, miR‐675, miR‐320b, miR‐378, miR‐328, miR‐23b‐3p) levels between VSMCs treated with high glucose or normal VSMCs, only miR‐34c level was obviously different between these groups

MicroRNAs (miRNAs) are non‐coding RNAs that function as regulators of numerous biological mechanisms, such as glucose metabolism and atherosclerosis. To search miRNAs potentially involved in the regulation of VSMCs proliferation and further interfere atherosclerosis, we measured and compared eight miRNAs (miR‐34c, miR‐144‐3p, miR‐224, miR‐675, miR‐320b, miR‐378, miR‐328, miR‐23b‐3p) expression profiles of VSMCs treated with high glucose or normal VSMCs using real‐time PCR. As shown in Figure 2, only miR‐34c was lowly expressed in cell treated with high glucose compared to that in normal control, and the other seven miRNAs expression levels in high glucose‐treated cells and normal control exhibited no significant change, indicated that miR‐34c expression was associated with atherosclerosis.

### 3.3 HMGB1 is a virtual target of miR‐34c

**Figure 3:**
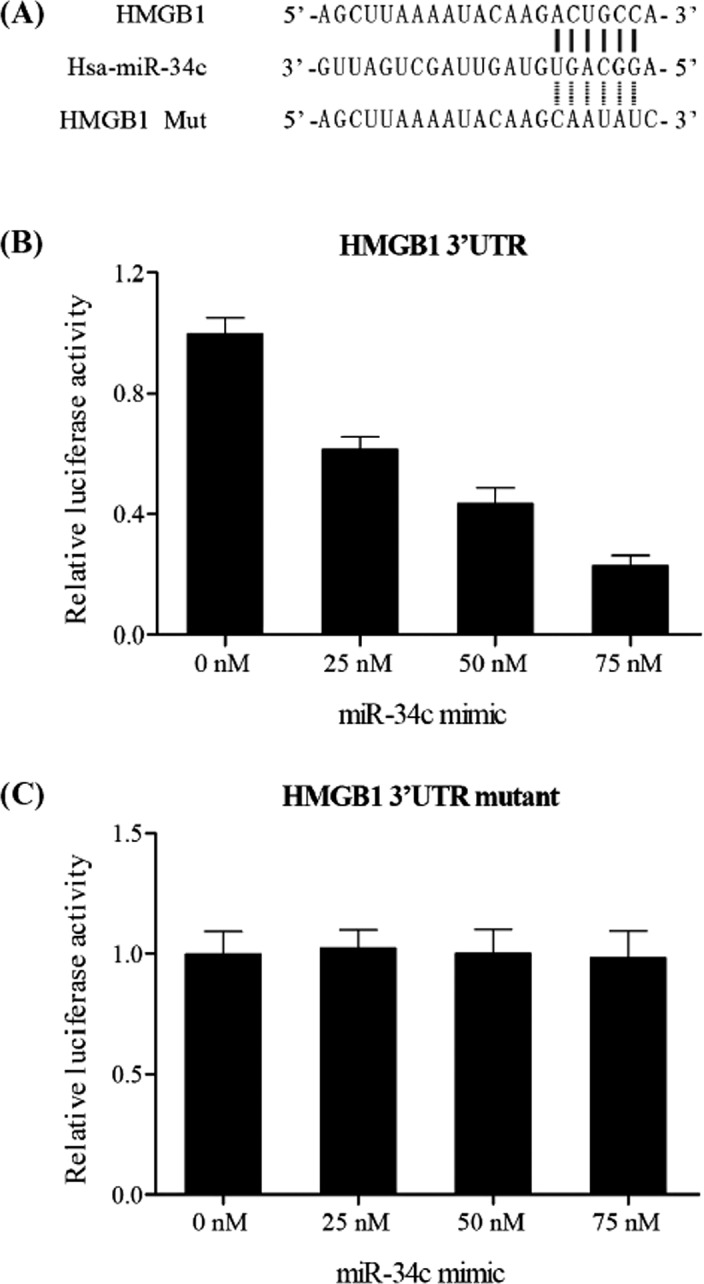
A, target of miR‐34c with predicted binding site resided in HMGB1 3′UTR. B, miR‐34c remarkably suppressed luciferase activity of wild HMGB1 3′UTR. C, Luciferase activities of the cells transfected with mutant HMGB1 3′UTR

It has been shown that miR‐34c was significantly associated with atherosclerosis, which might be responsible for the underlying molecular mechanism of miR‐34c. By using computational analysis, we identified that HMGB1 is a virtual target of miR‐34c with predicted binding site resided in HMGB1 3′UTR, as shown in Figure 3A. And, to abolish binding between HMGB1 3′UTR and miR‐34c, we mutated 6 bp nucleotides of HMGB1 that are paired to the seed sequence of miR‐34c with the use of site‐directed mutagenesis to generate mutant type HMGB1 3′UTR. In order to verify interactive between HMGB1 and miR‐34c, full length of wild HMGB1 3′UTR or mutant HMGB1 3′UTR was inserted into luciferase vector and co‐transfected with different dose of miR‐34c (0, 25, 50, 75 nmol/L). As shown in Figure 3B, miR‐34c remarkably suppressed luciferase activity of wild HMGB1 3′UTR under a concentration‐dependent manner in comparison with scramble control. However as shown in Figure 3C, luciferase activities of the cells transfected with mutant HMGB1 3′UTR and different dose of miR‐34c were comparable with each other. All data revealed that HMGB1 is a virtual target of miR‐34c with the binding site in HMGB1 3′UTR.

### 3.4 Various expressions of miR‐34c and HMGB1 in different treatments VSMCs

**Figure 4:**
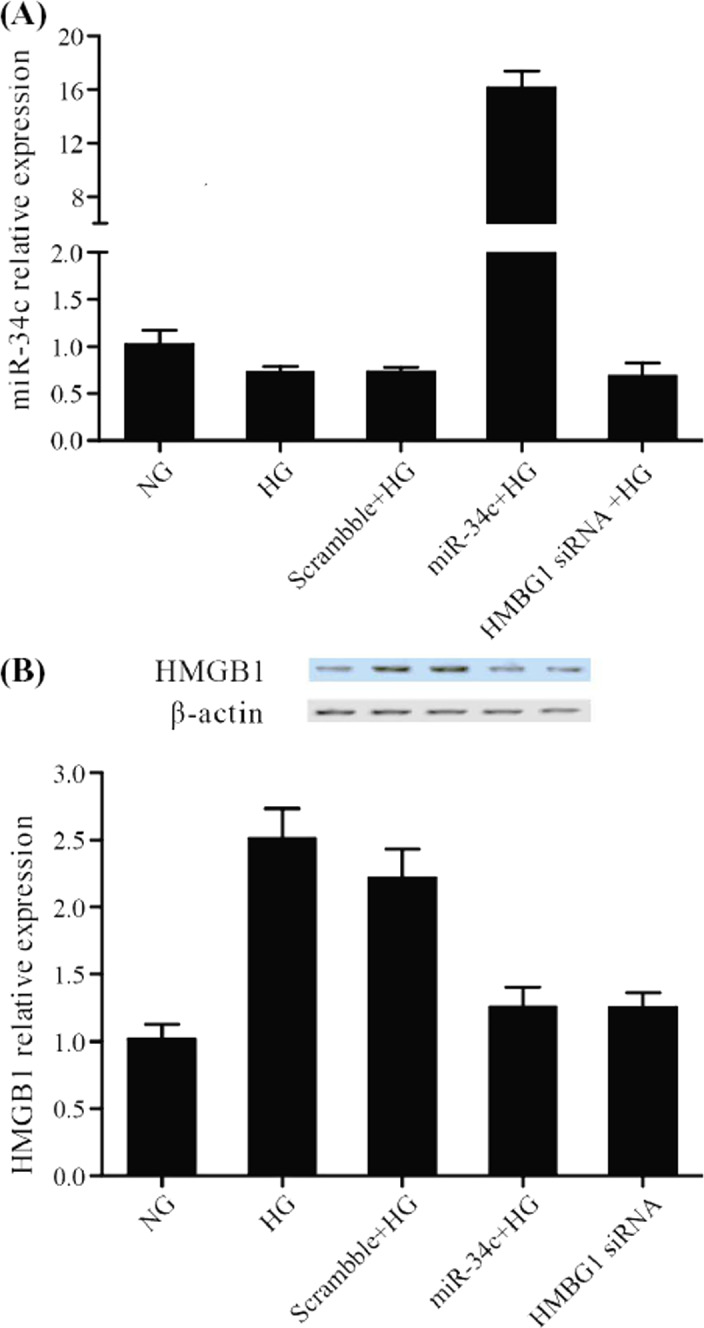
Real‐time PCR and Western blot were performed to detect effect of miR‐34c and HG on miR‐34c and HMGB1 levels. A, MiR‐34c remarkably increased miR‐34c expression. B, HG apparently increased HMGB1 level, but miR‐34c and HMGB1 siRNA remarkably decreased HMGB1 expression

To further explore whether high glucose (HG) and miR‐34c transfection affect miR‐34c and HMGB1 expression levels, real‐time PCR was performed to detect levels of miR‐34c and HMGB1 in normal VSMCs, or VSMCs treated with HG, or HG treated VSMCs transfection with miR‐34c, HMGB1 siRNA. As shown in Figure 4A, compared with normal VSMCs, cell‐treated HG showed no obvious difference. Moreover, compared with HG and HMGB1 siRNA treated VSMCs, miR‐34c remarkably up‐regulated miR‐34c expression. On the contrary, as shown in Figure 4B, glucose induced both mRNA and protein of HMGB1 highly expressed in VSMCs related to NC group, while transfection of miR‐34c and HMGB1 siRNA significantly reduced relative expressions of HMGB1 mRNA and protein. All results revealed that HG had no effect on miR‐34c and evidently up‐regulated HMGB1, and miR‐34c negative regulated HMGB1 level.

### 3.5 MiR‐34c suppressed high glucose caused proliferation of VSMC

**Figure 5:**
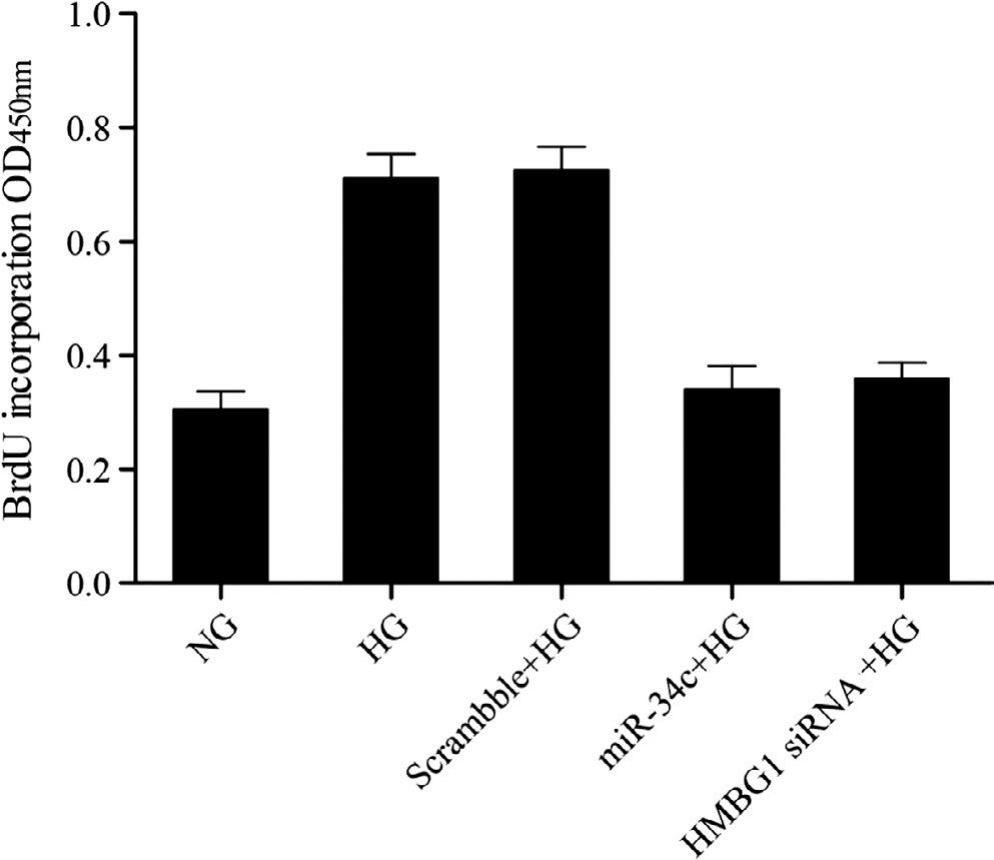
BrdU cell proliferation assay kit was utilized to measure effect of miR‐34c and HG on cell viability, and miR‐34c suppressed proliferation of VSMC caused by high glucose

It has been proved HG provoked proliferation of VSMCs (Figure 1); in following experiments, we tried to explore the role of miR‐34c on VSMCs viability using BrdU cell proliferation assay kit. As shown in Figure 5, after treating with HG, an evidently higher cell proliferation ability related to normal control was observed, the proliferation of VSMCs treated with HG apparently inhibited subsequently to transfect with miR‐34c and HMGB1 siRNA compared with scramble control, suggested that miR‐34c suppressed proliferation of VSMC caused by high glucose.

### 3.6 MiR‐34c reduced expressions of NF‐κB p56 and TNF‐α in VSMCs by high glucose

**Figure 6:**
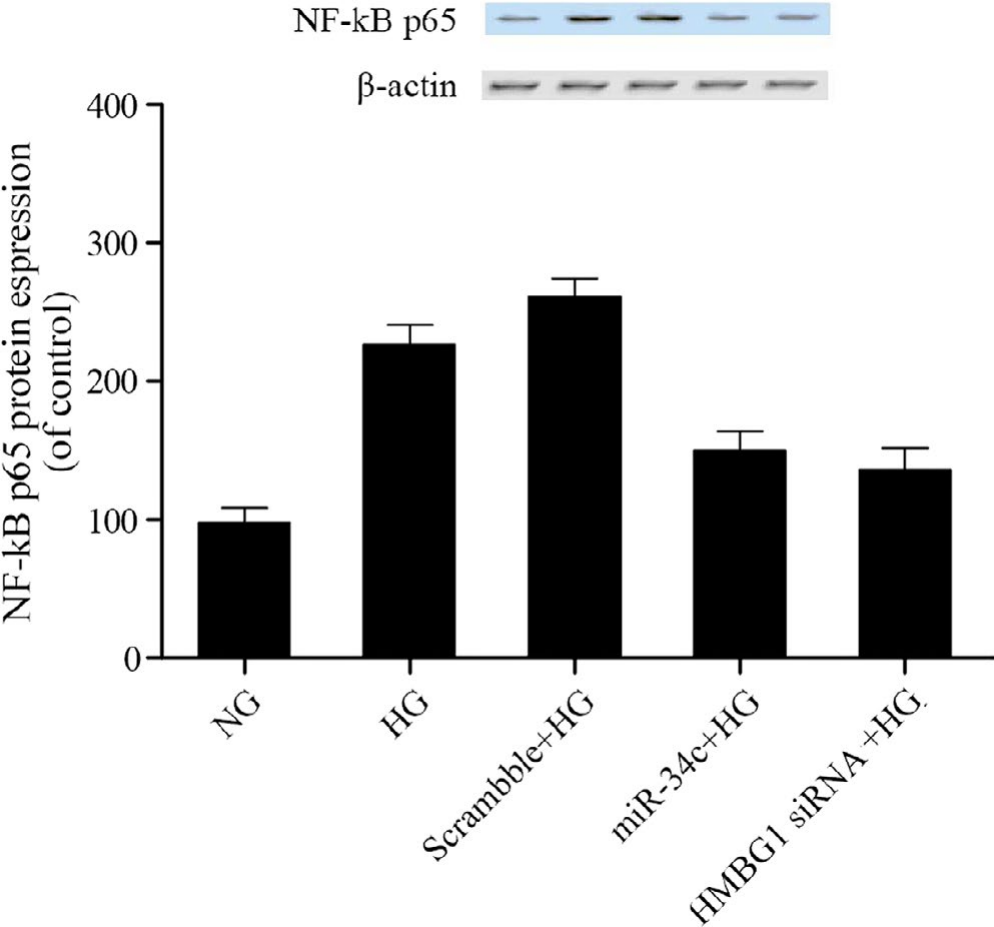
The influence of HG and miR‐34c on relative expressions of NF‐κB p65 was examined using Western blot analysis and real‐time PCR, and miR‐34c reduced expressions of NF‐κB p56 in VSMCs by high glucose

**Figure 7:**
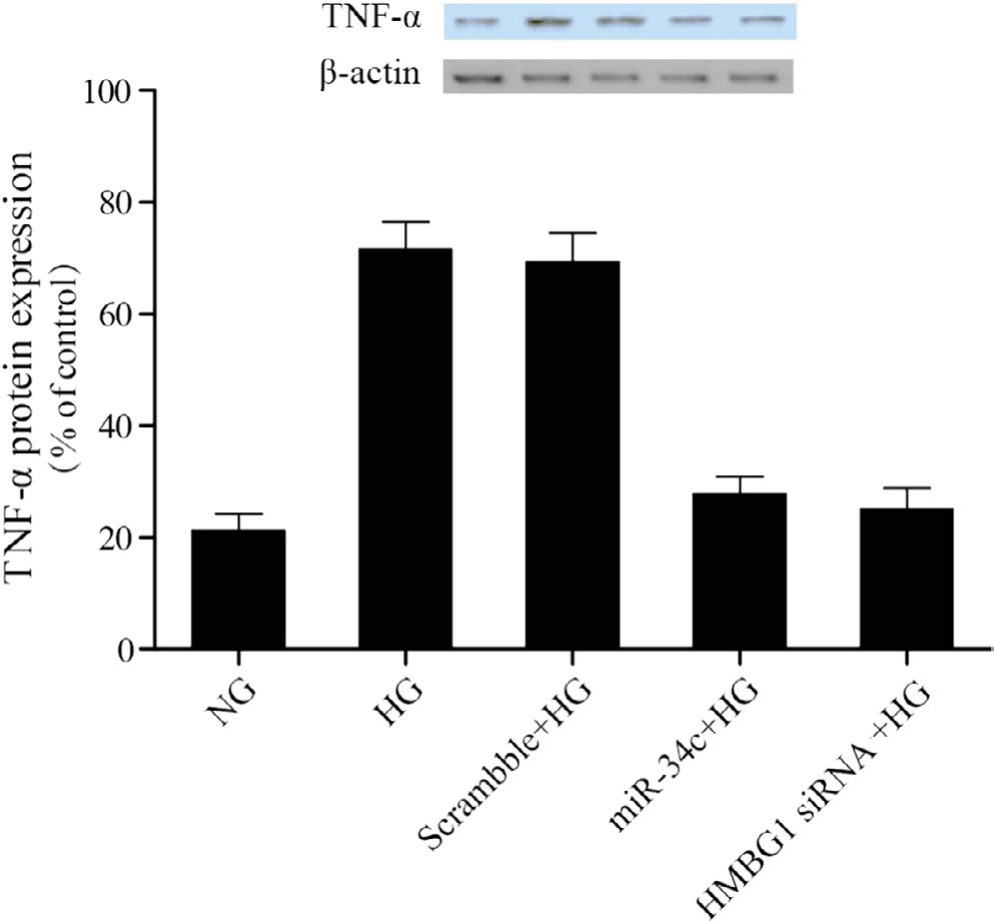
The impact of HG and miR‐34c on relative expressions of TNF‐α was measured by Western blot analysis and real‐time PCR, and miR‐34c reduced expressions of TNF‐α in VSMCs caused by high glucose

NF‐κB was proved to be a downstream signaling molecules of HMGB1, NF‐κB activation may play crucial roles in the mediation of viability of VSMCs and inflammatory processes, TNF‐α was one of pro‐inflammatory cytokines, so the relative expressions of NF‐κB p65 and TNF‐α were examined using Western blot analysis and real‐time PCR. As shown in Figures 6 and 7, NF‐κB p65 (Figure 6) and TNF‐α (Figure 7) mRNA and protein levels were increased following treating with HG in comparison with NC control; furthermore, transfection of miR‐34c and HMGB1 siRNA inhibited expressions of NF‐κB p65 (Figure 6) and TNF‐α (Figure 7) in VSMCs by high glucose, and there is no obvious difference on inhibitory effect of miR‐34c and HMGB1 siRNA. The data indicated that miR‐34C reduced expressions of NF‐κB p56 and TNF‐α in VSMCs by high glucose.

## 4 DISCUSSION

In this study, we performed BrdU assay to study whether glucose influenced VSMC proliferation and found that 5.5 and 10 mmol/L glucose had no obvious effect on VSMC proliferation, and glucose treatment up to 20 nmol/L, we observed an evidently higher cell proliferation ability related to normal control. Formation of neointima is often due to proliferation of VSMCs. Many studies have indicated the association between miRNAs and the activation of VSMC proliferation and migration. miR‐221, miR‐222, and miR‐21, in cultured rat VSMCs and rat balloon‐injured carotid arteries, were demonstrated to have regulatory roles in VSMC proliferation via B‐cell lymphoma 2 (Bcl‐2) and p27(Kip1), p57(Kip2), phosphatase and tensin homology (PTEN), respectively. Generally, miR‐146a is identified as an anti‐inflammatory gene in a variety of cells.^21^, ^22^ Sun et al further demonstrated that miR‐146a has a direct effect on Krupple‐like factor‐4 (KLF‐4) and revealed its key function in enhancement of VSMC proliferation in vascular neointimal hyperplasia and cultured rat VSMCs.^23^

MiRNAs triggers a range of cellular functions such as proliferation, differentiation, migration, and apoptosis.^24^, ^25^, ^26^, ^27^ Earlier reports have described that VSMCs in lesion formation and neointimal hyperplasia are regulated by miR‐221 and miR‐222, miR‐21, and miR‐145.^28^, ^29^ In this study, we then investigated whether eight miRNAs (miR‐34c, miR‐144‐3p, miR‐224‐SNP, miR‐675, miR‐320b, miR‐378, miR‐328, miR‐23b‐3p) associated with atherosclerosis using real‐time PCR and revealed that only miR‐34c level was associated with atherosclerosis with different expression between VSMCs treated with high glucose or normal VSMCs.

The specific mechanism of the role of miR‐34cin VSMC proliferation and atherosclerosis remains to be further studied while miR‐34c is associated with a variety of cellular processes in the cardiovascular system.^30^ The miR‐34 family (miR‐34a and b/c) is identified as trigger of the tumor inhibitory response of p53. The miR‐34a and b/c genes are activated by the transcription factor p53 responding to cellular stress, such as improper activation of oncogene, and a common characteristic of a variety of cancers is mutation in the p53 pathway.^31^, ^32^ The tumor suppressive role of miR‐34c includes suppression of migration by suppression of Met and Cav1; suppression of proliferation by suppression of E2f3 and Myb; and cell cycle arrest by suppression of Met, Cdk4, Ccne2, and c‐Myc.^33^ In this study, we performed in silico analysis and luciferase assay to understand the underlying molecular mechanism and found that HMGB1 is a candidate target of miR‐34c with predicted binding site located within HMGB1 3′UTR and also found that miR‐34c substantially and concentration‐dependently decreased luciferase activity of wild HMGB1 3′UTR, and miR‐34c cannot affect luciferase activity of mutant HMGB1 3′UTR.

High mobility group box protein 1, previously known as amphoterin or HMG1, is an extensive nuclear protein expressed in a lot of eukaryotic cells.^34^ It stabilizes nucleosomes and allows bending of DNA, which promotes gene transcription.^35^ HMGB1 has 2 typical and separate DNA‐binding domains, a 3‐hydroxy‐3‐methylglutaryl (HMG) A box and B box. Recently, surprising findings indicated that extracellularly released HMGB1 has cellular roles significantly different from its intranuclear roles.^36^ HMGB1 released from cells is a strong mediator of proliferation of VSMCs.^37^ As a highly conserved nuclear protein, HMGB1 is critical in a lot of pathogenic process such as aberrant proliferation and migration of VSMC.^38^ The limitation of our study was that more target of miR‐34c failure to study.

## 5 CONCLUSION

In summary, the findings of this present study demonstrated that HGMB1 is a direct target gene of miR‐34c, and miR‐34c might be a novel therapeutic strategy in the management of atherosclerosis by suppressing the expression of HGMB1, as well as its downstream pro‐inflammatory factor such TNF‐a and NF‐kB.
